# The feasibility and efficacy of a community-based multifactorial intervention to improve the cardiovascular risk factor control among patients with type 2 diabetes: A 2-year cluster randomized trial

**DOI:** 10.1097/MD.0000000000031943

**Published:** 2022-12-23

**Authors:** Wenhui Zhao, Yanlei Wang, Chenxiang Cao, Ziqiang Zeng, Lixia Jin, Zhaoxiang Liu, Min Duan, Yanan Dong, Jinpin Zhang, Ying Shuai, Na Wang, Yajing Zhang, Guixia Deng, Jiquan He, Xinghua Zhao, Wenli Zheng, Wenying Yang, Jianzhong Xiao, TelemedChina Study Group

**Affiliations:** a Beijing Tsinghua Changgung Hospital, School of Clinical Medicine, Tsinghua University, Beijing, China; b China-Japan Friendship Hospital, Beijing, China; c Beijing Pinggu District Hospital, Beijing, China; d Beijing Pinggu District Yukou Community Central Health Center, Beijing, China; e Beijing Pinggu District Xiagezhuang Township Hospital, Beijing, China; f Beijing Huairou District Yangsong Township Hospital, Beijing, China; g Beijing Daxing District Yinghai Community Central Health Center, Beijing, China.

**Keywords:** cardiovascular risk factor, community-based research/intervention, health insurance, primary care, type 2 diabetes

## Abstract

Our study is to investigate the feasibility and effectiveness of multiple cardiovascular factors intervention (MFI) in type 2 diabetes patients in China’s primary care setting.

**Methods:**

We performed a cluster randomized trial to compare the proportion of patients achieved the targets between usual care group (control, 9 sites, n = 868) and MFI group (8 sites, n = 739) among patients with type 2 diabetes in primary care setting. Logistic regression model with random effects was used to estimate the association of the effect of intervention and the proportion achieved the targets.

**Results:**

At baseline, the end of 1 year, and 2 years follow-up, the proportion of patients achieved all 3 target goals (HbA1c < 7.0%, blood pressure < 130/80 mm Hg and low-density lipoprotein cholesterol < 2.6 mmol/L) were 5.7%, 5.9%, 5.7% in the control group and 5.9%, 10.6%, 12.3% in the MFI group. After adjusting sex, age, diabetes duration, body mass index, HbA1c, blood pressure, and low-density lipoprotein cholesterol at baseline, there was no difference between the 2 groups (OR (95% CI): 1.27 (0.38–4.27) and 1.86 (0.79–4.38) for the first year and second year, respectively). When stratified by payment method, the patients with medical insurance or public expenses had a higher proportion achieved target goals (6.9% vs 16.4%, OR (95% CI): 2.30 (1.04–5.08)) in the second year.

**Conclusions:**

The controlling of cardiovascular risk factor targets remains suboptimal among patients with type 2 diabetes in primary care setting. MFI in type 2 diabetes improved cardiovascular disease risk profile, especially in the patients with medical insurance.

## 1. Introduction

Atherosclerotic cardiovascular disease (ASCVD) is the leading cause of death, hospital admission, and disability among people with type 2 diabetes, and the overall burden is expected to increase further as diabetes epidemic worldwide. According to the 3B studies of 2013,^[1]^ only 5.6% Chinese with diabetes achieved low-density lipoprotein cholesterol (LDL), blood pressure (BP), and glycosylated hemoglobin (HbA1c) control target which is much lower than in developed countries, for example, 21.2% of adults with diagnosed diabetes achieved all 3 risk factor control goals in United States.^[2]^ A targeted multiple cardiovascular factors intervention (MFI) including glucose control and other ASCVD risk factors substantially reduces ASCVD and got a median of 7.9 years of gain of life in people with type 2 diabetes.^[3]^ Evidences based diabetes guideline published and renewed every year. However, there is a wide gap between effective diabetes management practices and their implementation. Moreover, team-based care, care coordination, and integration of primary and specialty care have gained increasing attention and represent a promising area of development in chronic disease care in developing counties.^[4]^ Whether these evidences can be translated into real-life primary health care setting needs to be investigated. So we designed a primary care setting cluster randomized trial (clinical trial registered number ChiCTR-TRC-13003222) that compared the usual clinical practice with a protocol-driven treatment strategy aimed at the optimal correction of major ASCVD risk factors in patients with type 2 diabetes. The aim was to evaluate the feasibility and efficacy of MFI assigned according to guidelines for global ASCVD risk factor control in people with type 2 diabetes in the primary care setting.

## 2. Research design and methods

### 2.1. Study population

Our study was designed to be a 3-arm community clinics-cluster randomized controlled trial, control and MFI and the exercise intervention group. The data of the exercise intervention group cannot be collected for the failure of the exercise monitor. 17 community clinics with a ratio 1: 1, were randomized into 2 groups, usual care group therapy (Control group) and MFI group by a computer assisted software. The participants inclusion criteria were all type 2 diabetes patients aged 20 to 80 years; not achieved all 3 target goals (HbA1c < 7.0%, BP < 130/80 mm Hg or LDL < 2.6 mmol/L). The exclusion criteria were patients with severe cardiovascular, liver, or kidney diseases; end-stage cancers; pregnant; any reason that the investigators think the subject should not be enrolled.

### 2.2. Methods

#### 2.2.1. Intervention procedures.

Our protocol-driven treatment strategy includes the community clinics doctor training and the patient intensive management in the MFI group. The intervention was implemented by a hierarchy care assignment, including 5 elements: an internet-based interactive-information platform, in which doctors in the community clinics and experts in the tier 2 and tier 3 hospital were able to access the electronic medical records; a computer-assisting multiple ASCVD risk factors management. For every patient, multiple ASCVD risk factors especially hyperglycemia, hypertension and dyslipidemia were asked to be managed. As compared to the target goal of Chinese Diabetes Society guideline, the gap was asked to narrow by adjusting therapy regimens during the follow-up. The indication of aspirin and statin was reviewed for every visit to community clinics and prescribed if necessary. A share care was given by both local community doctors and experts from tier 2 and/or tier 3 hospital. Routine management was given by general doctors in community clinics. The treatment regimens were reviewed individually by both community doctors and experts. Every doctor in community managed up to 100 patients, and every expert was responsible for 2 community clinics. Those complicated cases were consulted to the experts from tier 2 and tier 3; an examination of HbA1c, BP, and lipid profile was performed every 3 months in the first year, and every 6 months in the second year; laboratory tests and complication screening examinations offered by local tier 2 and/or tier 3 hospitals provided those were not available in the community clinics. Usual care was given for those patients in the control group and checkup at baseline and yearly.

#### 2.2.2. The evaluation of patients.

Diagnostic criteria for diabetes were employed as described by the World Health Organization in 1999. All patients signed an informed consent prior to data collection. A standard questionnaire was administered by doctors in community clinics included questions related to the personal education, family income, medical insurance status, medical history, and current treatment. Body weight, height, waist circumference, BP measured, and 10 g nylon filament sensation examination was performed; lipid profile, HbA1c, urine albumin/creatinine ratio, liver enzyme, and serum creatinine were determined; fundus examination was performed and digital fundus photography was taken and reviewed by oculist, ankle/brachial index and carotid artery ultrasonography were carried out. For each patient, the ASCVD risk factors, including smoking status, body mass index (BMI), BP, LDL, HbA1c, and screening for microvascular complications and macrovascular complications yearly. This analysis is based on the results from 2-year follow-up.

#### 2.2.3. Outcomes.

HbA1c < 7%, BP < 130/80 mm Hg, and low-density lipoprotein cholesterol (LDL) < 2.6 mmol/L were set as target goals. Achievement of glycated hemoglobin, BP, and low-density lipoprotein cholesterol (ABC) target goal listed as above at the end of 2 years after baseline was primary outcome and the end of 1 year as second outcome.

### 2.3. Statistical analysis

Standard descriptive statistics [mean and standard deviation for normal continuous variable, median, and interquartile range for skewed continuous one, while frequencies and percentages per category for categorical one] were used to demonstrate baseline characteristics of patients in each group (intervention vs control).

Efficacy analyses were performed primarily on the full analysis set by the intention-to-treat method. Full analysis set included all subjects who were randomized to study, received intervention more than one times and contributed at least one primary efficacy value after baseline. Last observation carried forward were applied to handle missing data of HbA1c, BP, and LDL. All other missing data were not imputed. No interim analysis was conducted.

The numbers and percentages of patients achieving each efficacy end point were reported by group. Considering the cluster design, logistic regression with random effects (LRRE) was employed in our analysis due to its ability to taken between cluster variation into account.^[5]^ We first fit univariate LRRE models to evaluate the effects of intervention on different efficacy endpoints at years 1 and 2. The results were presented as odds ratios (ORs) with 95% confidence intervals (CIs). Then, bivariate LRRE models corrected for the baseline performances of the specific efficacy endpoint were conducted to get the adjusted ORs and corresponding 95% CIs among the 2 groups.

To further explore the effect of intervention on primary endpoint, we performed multivariate LRRE adjusted for sex (male vs female), age (≤ 60 vs > 60), diabetes duration (≤ 5 vs. 6–10 vs > 10), BMI (< 24 vs 24–28 vs ≥ 28), HbA1c (< 7.5 vs ≥ 7.5), BP (< 140/90 vs ≥ 140/90), and LDL (≤ 3.1 vs > 3.1) at baseline as the post hoc sensitivity analysis.

The statistical significance is defined as the 2-sided *P* value less than 0.05. All analyses were performed by SAS software, Version 9.4 of the SAS System for Windows. Copyright ^©^ 2013 SAS Institute Inc.

### 2.4. Ethics

This study was approved by the Ethic Committee of China-Japan Friendship Hospital (No.2013-7,2013-8).

## 3. Results

There were 1607 patients (868 in control group and 739 in intervention group) enrolled in this study. In the end of 1- and 2-year follow-up, 767 (88.4%) and 725 (83.5%) in control group, 644 (87.1%) and 617 (83.5%) in MFI group completed yearly follow-up. Five patents in the control group died in the first-year follow-up. And 4 and 1 patient in the MFI group died in the first- and second-year follow-up. Most demographic and clinical parameters were comparable at baseline except for higher proportion of poor insurance, current smoker, lower education levels, and shorter diabetes duration in the control group (Table 1). At baseline, the proportion of patients achieved all 3 target goals (HbA1c < 7.0%, BP < 130/80 mm Hg and LDL < 2.6 mmol/l) were 5.7% and 5.9% in the control group and the MFI group. MFI largely increased the proportion of people achieving all 3 ABC goals in the end of the first and second year (10.6% vs 5.9% and 12.3% vs 5.7%) compared with control group, while other characteristics did not change much (Table 2). MFI largely increased the proportion of people reached target goal of BP and a little improvement of LDL level, but not the HbA1c level (Table 3).

**Table 1:** Baseline characteristics of control group and intervention group.

	Control group (N = 868)	Intervention group (N = 739)	ICC
Age (yr)			
≤60	389 (44.8%)	367 (50.0%)	
>60	479 (55.2%)	367 (50.0%)	
Median (P25, P75)	61.1 (54.6, 67.1)	60.0 (53.5, 66.1)	
Sex			0.014
Male	328 (37.8%)	304 (41.1%)	
Female	540 (62.2%)	435 (58.9%)	
Education*			0.165
Junior high school or below	599 (69.5%)	403 (54.7%)	
Senior high school	181 (21.0%)	240 (32.6%)	
Undergraduate or above	82 (9.5%)	94 (12.8%)	
Missing	6	2	
Payment method			0.516
Medical insurance	475 (55.5%)	506 (70.1%)	
Public expenses	88 (10.3%)	4 (0.6%)	
Personal payment	283 (33.1%)	206 (28.5%)	
Others	10 (1.2%)	6 (0.8%)	
Missing	12	17	
Diabetes duration (yr)			
≤5	385 (44.4%)	310 (42.3%)	
6-10	236 (27.2%)	178 (24.3%)	
>10	246 (28.4%)	245 (33.4%)	
Median (P25, P75)	5.6 (2.6, 10.4)	6.1 (3.2, 10.9)	
Missing	0	1	
HbA1c (%)			0.045
Median (P25, P75)	7.0 (6.3, 8.1)	7.0 (6.3, 7.9)	
HbA1c (%)			0.038
<7	393 (45.3%)	364 (49.3%)	
≥7	475 (54.7%)	375 (50.7%)	
HbA1c (%)			0.035
≤7.5	567 (65.3%)	496 (67.1%)	
7.5–9	182 (21.0%)	144 (19.5%)	
>9	119 (13.7%)	99 (13.4%)	
SBP (mm Hg)			0.110
Median (P25, P75)	130.0 (120.0, 134.0)	130.0 (120.0, 135.0)	
DBP (mm Hg)			0.114
Median (P25, P75)	80.0 (76.0, 80.0)	80.0 (75.0, 80.0)	
BP (mm Hg)			0.159
SBP < 130 and DBP < 80	151 (17.4%)	167 (22.6%)	
SBP ≥ 130 or DBP ≥ 80	717 (82.6%)	572 (77.4%)	
BP (mm Hg)			0.081
SBP < 140 and DBP < 90	662 (76.3%)	588 (79.6%)	
140 ≤ SBP < 160 or 90 ≤ DBP < 100	165 (19.0%)	131 (17.7%)	
SBP ≥ 160 or DBP ≥ 100	41 (4.7%)	20 (2.7%)	
LDL (mmol/L)			0.096
Median (P25, P75)	2.8 (2.3, 3.4)	2.7 (2.2, 3.4)	
LDL (mmol/L)			0.047
<2.6	314 (36.2%)	327 (44.2%)	
≥2.6	554 (63.8%)	412 (55.8%)	
LDL (mmol/L)			0.088
≤3.5	685 (78.9%)	594 (80.4%)	
3.5-4.5	121 (13.9%)	84 (11.4%)	
>4.5	62 (7.1%)	61 (8.3%)	
BMI (kg/m^2^)			0.022
Median (P25, P75)	25.6 (23.5, 27.8)	25.8 (23.7, 27.9)	
BMI (kg/m^2^)			0.022
<24	255 (29.4%)	207 (28.0%)	
24–28	405 (46.7%)	351 (47.5%)	
≥28	208 (24.0%)	181 (24.5%)	
Waist (cm)			0.062
Median (P25, P75)	87.0 (82.0, 92.0)	89.0 (84.0, 95.0)	

* Senior high school including technical secondary college; undergraduate including junior college.

**Defined as reaching 2 of the following targets: HbA1c < 7%, BP < 130/80 mm Hg, and LDL < 2.6 mmol/L.

***Defined as reaching at least one of the following targets: HbA1c < 7%, BP < 130/80 mm Hg, and LDL < 2.6 mmol/L.

BMI = body mass index, BP = blood pressure, CI = confidence interval, DBP = diastolic blood pressure, HbA1c = hemoglobin A1c, LDL = low density lipoprotein, P25 = 25th percentile, P75 = 75th percentile, SBP = systolic blood pressure.

**Table 2:** Year 1 and year 2 characteristics of control group and intervention group.

	Control group	Intervention group
At year 1		
LDL (mmol/L) median (P25,P75)	2.7 (2.2,3.2)	2.6 (2.1,3.2)
HbA1c (%) median (P25,P75)	7.0 (6.3,8.1)	7.0 (6.3,8.1)
SBP (mm Hg) median (P25,P75)	130.0 (120.0,140.0)	128.0 (120.0,133.0)
DBP (mm Hg) median (P25,P75)	80.0 (70.0,82.0)	78.0 (73.0,80.0)
Weight (kg) median (P25,P75)	69.0 (62.0,75.0)	68.0 (62.0,75.0)
Waist (cm) median (P25,P75)	87.0 (82.0,94.0)	88.0 (84.0,94.0)
BMI (kg/m^2^) median (P25,P75)	25.8 (23.6,28.0)	25.6 (23.7,27.6)
At year 2		
LDL(mmol/L) median (P25,P75)	2.7 (2.2,3.2)	2.6 (2.1,3.2)
HbA1c (%) median (P25,P75)	6.8 (6.1,7.9)	6.9 (6.2,7.9)
SBP (mm Hg) median (P25,P75)	130.0 (120.0,138.0)	130.0 (120.0,132.0)
DBP (mm Hg) median (P25,P75)	80.0 (72.0,82.0)	78.0 (73.0,80.0)
Weight (kg) median (P25,P75)	69.0 (62.0,75.0)	68.0 (62.0,75.0)
Waist (cm) median (P25,P75)	87.0 (81.0,93.0)	88.0 (84.0,95.0)
BMI (kg/m^2^) median (P25,P75)	25.8 (23.7,28.0)	25.5 (23.7,27.7)

BMI = body mass index, DBP = diastolic blood pressure, HbA1c = hemoglobin A1c, LDL = low density lipoprotein, P25 = 25th percentile, P75 = 75th percentile, SBP = systolic blood pressure;

**Table 3:** Comparisons of efficacy endpoints between control and intervention group at year 1 and year 2 after baseline.

	N/Total N (%)		Odds ratio (95%CI)*			
	Control group	Intervention group	Unadjusted	Adjusted§	ICC	*P* value#
At baseline						
Reaching combined control target**						
Yes	0/868 (0.0%)	0/739 (0.0%)	NA	NA	0.049	.854
Reaching at least 2 targets***			NA	NA		
Yes	242/868 (27.9%)	276/739 (37.3%)	NA	NA	0.050	<.001
Reaching at least 1 target****			NA	NA		
Yes	616/868 (71.0%)	582/739 (78.8%)	NA	NA	0.044	<.001
HbA1c (%)			NA	NA		
<7	393/868 (45.3%)	364/739 (49.3%)	NA	NA	0.038	.111
BP (mm Hg)			NA	NA		
<130/80	151/868 (17.4%)	167/739 (22.6%)	NA	NA	0.159	.009
LDL (mmol/L)			NA	NA		
<2.6	314/868 (36.2%)	327/739 (44.2%)	NA	NA	0.096	.061
At year 1 after baseline						
Reaching combined control target**						
Yes	44/748 (5.9%)	68/642 (10.6%)	1.43 (0.42–4.89)	1.43 (0.42–4.89)	0.098	.565
Reaching at least 2 targets***						
Yes	234/748 (31.3%)	296/642 (46.1%)	1.90 (0.83–4.34)	1.72 (0.72–4.09)	0.155	.220
Reaching at least 1 target****						
Yes	574/748 (76.7%)	518/642 (80.7%)	1.37 (0.57–3.28)	1.18 (0.51–2.73)	0.066	0.692
HbA1c (%)						
<7	366/755 (48.5%)	296/642 (46.1%)	0.91 (0.61–1.34)	0.80 (0.49–1.29)	0.026	.360
BP (mm Hg)						
<130/80	159/760 (20.9%)	268/644 (41.6%)	3.28 (0.90–12.01)	3.23 (0.95–10.93)	0.270	.060
LDL (mmol/L)						
<2.6	344/755 (45.6%)	318/642 (49.5%)	1.17 (0.71–1.92)	1.05 (0.60–1.83)	0.049	.860
At year 2 after baseline						
Reaching combined control target**						
Yes	41/721 (5.7%)	76/616 (12.3%)	2.14 (0.84–5.44)	2.14 (0.84–5.44)	0.060	.110
Reaching at least 2 targets***						
Yes	229/721 (31.8%)	281/616 (45.6%)	1.72 (0.90–3.29)	1.61 (0.83–3.10)	0.099	.158
Reaching at least 1 target****						
Yes	573/721 (79.5%)	497/616 (80.7%)	1.14 (0.56–2.31)	1.02 (0.51–2.02)	0.049	.964
HbA1c (%)						
<7	392/725 (54.1%)	309/617 (50.1%)	0.84 (0.49–1.44)	0.77 (0.41–1.47)	0.054	.430
BP (mm Hg)						
<130/80	137/721 (19.0%)	231/617 (37.4%)	2.55 (0.71–9.22)	2.52 (0.74–8.57)	0.229	.138
LDL (mmol/L)						
<2.6	320/725 (44.1%)	316/616 (51.3%)	1.30 (0.92–1.83)	1.18 (0.79–1.77)	0.022	.426

BP = blood pressure; CI = confidence interval; HbA1c = hemoglobin A1c; ICC = intracluster correlation coefficient; LDL = low-density lipoprotein.

* Calculated based on the logistic regression model with random effects.

§ Adjusted the value of the outcome at baseline.

#*P* value for the logistic regression model with random effects adjusted the value of the outcome at baseline.

** Defined as reaching HbA1c < 7%, BP < 130/80 mm Hg, and LDL < 2.6 mmol/L simultaneously.

*** Defined as reaching at least 2 of the following targets: HbA1c < 7%, BP < 130/80 mm Hg, and LDL < 2.6 mmol/L.

*** *Defined as reaching at least one of the following targets: HbA1c < 7%, BP < 130/80 mm Hg, and LDL < 2.6 mmol/L.

Logistic regression model with random effects was used to estimate the association of the effect of intervention and the proportion achieved the targets. After adjusting sex, age, payment method, diabetes duration, BMI, HbA1c, BP and LDL at baseline, there was no difference between the 2 groups (OR (95%CI): 1.25 (0.37-4.21) and 1.92 (0.82-4.47) for the first year and second year, respectively) (table 4).

**Table 4:** Multiple logistic regression with random effect for primary efficacy endpoints at year 1 and year 2 after baseline.

	N/Total N (%)		Odds ratio (95%CI)*	
	Control group	Intervention group		*P* value
At year 1 after baseline				
Reaching combined control target**				
Yes	44/748 (5.9%)	68/642 (10.6%)	1.24 (0.37–4.18)	.726
At year 2 after baseline				
Reaching combined control target**				
Yes	41/721 (5.7%)	76/616 (12.3%)	1.92 (0.82–4.47)	.131

BP = blood pressure, CI = confidence interval, HbA1c = hemoglobin A1c, ICC = intracluster correlation coefficient, LDL = low density lipoprotein.

* Calculated based on the logistic regression model with random effects adjusting for sex, age, diabetes duration, payment method, BMI, HbA1c, BP and LDL at baseline.

** Defined as reaching HbA1c < 7%, BP < 130/80 mm Hg, and LDL < 2.6 mmol/L simultaneously.

When stratified by payment method, the tests for interaction were not statistically significant, which indicated that the intervention effects were consistent across different payment method group. (table 5).

**Table 5:** Multiple logistic regression with random effect for primary efficacy endpoints at year 1 and year 2 after baseline stratified by payment method.

	N/Total N (%)		Odds ratio (95%CI)*		
	Control Group	Intervention Group		*P* value	*P* for interaction
At year 1 after baseline					
Reaching combined control target**					.439
Yes (patients with medical insurance or public expenses)	36/426 (8.5%)	60/442 (13.6%)	0.95 (0.29–3.14)	.935	
Yes (patients with personal payment or others)	8/319 (2.5%)	6/188 (3.2%)	1.49 (0.30–7.34)	.624	
At year 2 after baseline					
Reaching combined control target**					.165
Yes (patients with medical insurance or public expenses)	28/405 (6.9%)	70/427 (16.4%)	2.30 (1.04-5.08)	.040	
Yes (patients with personal payment or others)	12/313 (3.8%)	5/178 (2.8%)	0.74 (0.17-3.18)	.689	

BP = blood pressure, CI = confidence interval, HbA1c = hemoglobin A1c, ICC = intracluster correlation coefficient, LDL = low density lipoprotein.

* Adjusted the value of the outcome at baseline.

** Defined as reaching HbA1c < 7%, BP < 130/80 mm Hg and LDL < 2.6 mmol/L simultaneously.

## 4. Discussion

In this cluster randomized trial, we found MFI was feasible but there was no statistical difference between the 2 groups with the improvement in ASCVD risk profile in the real-life primary health care setting. While when stratified by payment method, the patients with medical insurance or public expenses might have a higher proportion achieved target goals in the second year.

ASCVD is the leading cause of death, hospital admission, and disability among people with type 2 diabetes, and it is expected to increase the further overall burden as the result of a worldwide diabetes epidemic. Numerous studies have demonstrated positive association between MFI involving glucose control and the correction of multiple ASCVD risk factors and the risk of ASCVD and all-cause mortality in people with type 2 diabetes.^[6,7]^ Likewise, an observational study published in 2013 showed that patients with type 2 diabetes who had uncontrolled BP, LDL, and HbA1c or with only their HbA1c at target, were at greatest risk of hospitalization for cardiovascular disease, whereas those with all 3 risk factors controlled or with BP and LDL at target had lower rates of adverse cardiovascular events.^[8]^ In the Steno-2 study, a MFI milestone study, showed about 50% reduction in the risk of cardiovascular events.^[3]^ But in real world, the controlling of cardiovascular risk factor targets remains suboptimal among patients with type 2 diabetes, especially in the developing countries, i.e., only 5.6% achieved LDL, BP, and HbA1c control target in China,^[1]^ which is much lower than in America, which is 21.2% of adults with diagnosed diabetes achieved all 3 risk factor control goals in 2015–2018.^[2]^ Identifying barriers of target achievement is worthy of establishing better strategy both for care providers and government.

The mechanisms of why multiple cardiovascular factors intervention improve ASCVD outcome maybe at least partly related to inflammatory burden amelioration. Sodium-dependent glucose transporters 2 inhibitors improves HbA1c, BP, LDL control, and body weight loss.^[9]^ Weight loss, especially fat tissue loss is associated with the decreasing of cytokine like Neuregulin-4 in diabetics with lower HbA1c levels.^[10,11]^ But we did not measure inflammatory markers in this study.

Community-based strategies in the prevention and control of diabetes have gained increasing attention and represent a promising area of development in chronic disease care in developing counties^[12–14]^ and are more cost-effective compared with outpatient specialists.^[15]^ But in China, such conclusive evidence is of absence in China, community-based cluster randomized trial studies might provide useful information to clarify this question.

In this study, 1607 patients (868 in control group and 739 in intervention group) were enrolled with 1- and 2-year follow-up rate of 88.4% and 83.5% in control group, 87.1% and 83.5% in MFI group, respectively. At baseline and during the follow-up of 2 years, the proportion of patients achieved all 3 target goals were 5.9%, 5.7% in the control group and 10.6% 12.3% in the MFI group. The intervention showed improved outcomes over 2 years, but there was no significant difference compared with the control group. After adjusting sex, age, diabetes duration, BMI, HbA1c, BP, and LDL at baseline, there was no difference between the 2 groups (OR (95%CI): 1.27 (0.38–4.27) and 1.86 (0.79–4.38) for the first year and second year, respectively. An explain is the limitation of trial design, cluster randomization should include a large cluster number but a small group size. There was another study^[16]^ showed the multifaceted primary health care intervention did not improve ASCVD risk management in participating Australian general practices which show the difficulty of implementing complex quality improvement in primary care setting.

We found an improved target goals rate in the population with medical insurance or public expenses with a higher proportion achieved target goals (6.9% vs 16.4%, OR (95% CI): 2.30 (1.04–5.08)) in the second year. Further studies with a large cluster size are needed to clarify these findings. The good news for diabetes patients is price for generic medicine including stains, antihypertensive medicine and hypoglycemic agents are markedly decreased in China. Commonly used drugs for hypertension, hyperglycemia, and hypercholesterolemia only cost about one-tenth of previous price. This strategy largely improves the affordability for patient with noninfectious chronic diseases like diabetes.

In this cluster randomized trial, we found the hierarchy care basing MFI was feasible. MFI had a tendency to increase the proportion of people achieving all 3 ABC goals in the end of the first and second year (10.6% vs 5.9% and 12.3% vs 5.7%) compared with control group. However, due to the cluster randomized design and variation among various communities, namely the intracluster variation, the difference was not significant. We found that no improvement found in those community clinics in rural area without good medical insurance in MFI group. In contrast, those assigned to control group with good medical insurance support had a significant improvement for global ASCVD risk management. When stratified by payment category, MFI significant increase the proportion of patients attained all 3 targets, i.e., the patients with medical insurance or public expenses have a higher proportion achieved target goals in the second year. The improvement was obviously when compared with baseline data in MFI group, i.e., the proportion of patient attained 3 target goal increased from 5.9% at baseline to 12.3% after 2 years intervention. The results of a study from Massachusetts healthcare reform suggest that increasing publicly subsidized health insurance had a positive impact on primary care access for lower income adults.^[17]^ Combined the result of our study, we argued that this hierarchy care based, MFI would be effective given patents had a better medical insurance in the primary care setting of China.

In this study, more than half patients achieved HbA1c target goal but did not change from baseline. In contrast, the proportion of patients reached BP and LDL target increased. Among these 3 ASCVD risk factors, BP and LDL control was significantly associated with reduced ASCVD hospitalization risk, especially when both risk factors were well controlled.^[16]^ Another study from China showed among those achieving only 1 ABC target, LDL reduction was associated with the greatest ASCVD risk reduction (42%), followed by BP reduction (18%), and HbA1c reduction (13%).^[18]^ BP and LDL seem to be are easier to be controlled than hyperglycemia, suggesting that strategy should focus on the control of BP and LDL.

The strengths of this study included the long follow-up period (2 years), and recruitment from a primary care setting, thus increasing the generalizability of our findings. This study had several potential limitations include unblinded assignment, small number of clusters, no mandatory adjustment for treatment regimen, and no inflammatory markers measured. The study was performed in a regional population may not be representative of those experienced by the general population. The analysis did not include the data of the exercise intervention group for the failure of the exercise monitor. Based on the limitation above, our findings may need further confirmed by longitudinal studies with a large cluster size.

In summary, our study demonstrated that the hierarchy cared based MFI improved outcomes over 2 years only in those people with good medical insurance support. New price policy for generic medicine in China would improve the affordability and finally improve the poor control status of ASCVD risk factors in patients with diabetes.

## Author contributions

**Data curation:** Chenxiang Cao, Ziqiang Zeng.

**Formal analysis:** Yanlei Wang.

**Investigation:** Lixia Jin.

**Resources:** Zhaoxiang Liu.

**Writing—original draft:** Wenhui Zhao.
